# Age-related changes in migratory behaviour within the first annual cycle of a passerine bird

**DOI:** 10.1371/journal.pone.0273686

**Published:** 2022-10-19

**Authors:** Robert Patchett, Alexander N. G. Kirschel, Joanna Robins King, Patrick Styles, Will Cresswell

**Affiliations:** 1 Centre for Biological Diversity, University of St Andrews, St Andrews, United Kingdom; 2 Department of Biological Sciences, University of Cyprus, Nicosia, Cyprus; MARE – Marine and Environmental Sciences Centre, PORTUGAL

## Abstract

First time migrants (juveniles hereafter) of many species migrate without specific knowledge of non-breeding locations, but experience may aid adults in timing and route decisions because they can migrate more efficiently to their previous non-breeding sites. Consequently, we expect a transition to more efficient migratory behaviour with age, but when and how this happens is little known. We used light-level geolocator data from Cyprus wheatears *Oenanthe cypriaca* to compare migration timing and route directness between juveniles and adults, and repeatability of their timing and non-breeding locations. We predicted that juveniles would depart and arrive later than adults for both autumn and spring migration; that duration of migration would be greater for juveniles; that routes taken by juveniles would be less direct than those for adults; and that autumn and spring departure timing, and non-breeding locations, would be more repeatable for adults between two years than for juveniles between their first and subsequent migration. We found that juveniles departed significantly later than adults in autumn but there was no difference in arrival timing, and although spring departure timings did not differ, juveniles arrived on the breeding grounds later than adults. Nevertheless, we found no significant age-related difference in the duration of migration in autumn or spring. Yet, juvenile migrations were less direct than those of adults in autumn, but not spring. We found evidence that spring departure timing and non-breeding locations were repeatable for adults but not juveniles. Our findings show that age-related changes in migratory behaviour begin to occur during the first annual cycle demonstrating the potential for early adaptation to environmental variability within an individual’s life.

## Introduction

Migratory animals make annual movements to gain fitness advantages from matching their movements to variable resources in space and time [1]. But individuals migrating for the first time (juveniles hereafter) in many species, unless they accompany adults, must decide when to leave and what route to take without any prior experience [2]. Consequently, this first migration can be a particularly risky period in an individual’s life [3, 4], with mortality during migration an important factor in population dynamics [5]. Age-related differences in migration are also important in understanding the evolution of migration and how species might adapt to environmental change [6]. For example, the stochasticity inherent in juvenile migrations [e.g. 7] means that some birds are likely to end up in sub-optimal non-breeding locations, but others may end up in newly suitable habitat as it shifts with variable climate, leading to differential survival and thus conferring population-level resilience to change [8–10]. Furthermore, how flexible individuals are in their migration timing [e.g. 11] and non-breeding locations between years [e.g. 12] is important in understanding resilience to change at the individual level. Despite its clear importance, we lack knowledge of how experience affects flexibility in individual migration and non-breeding site use for most migrant species that are too small to carry satellite or other large non-archival tags.

The endogenous migration program in birds can determine direction, distance and timing of migration [13, 14]. In addition, adult migrants are experienced, which may aid them in following their previous route and in locating previously visited stopovers and non-breeding sites [15]. Juveniles, however, have no experience of migration and in many species presumably depend on their endogenous program [2]. But in some species (e.g. geese, swans and cranes) social learning is crucial in learning their migration route and non-breeding destination [16, 17].

These differences between first time migrants and experienced birds are often reflected temporally and spatially. Departure timing varies, with examples of adults leaving before juveniles [18, 19] and vice versa [20]. Juveniles may spend longer at stopovers than adults [21, 22] and sometimes take them more frequently [18]. Spatial differences occur, with migration routes of first-time migrants often being less direct than those of adults [21], with juveniles less likely to correct for wind drift [23] and more likely to be displaced [2]. Finally, adults in many species show high site fidelity to non-breeding sites [2, 8, 24–26], particularly for more generalist foraging species [27]. Juveniles of these species are likely to disperse to some extent until they find a suitable first non-breeding territory [e.g. 28] perhaps at a relatively small scale [8], although there are currently no studies of repeatedly tracked juvenile passerines tagged on the non-breeding grounds that allow this process to be observed.

Age-related differences in migration may also depend on sex because male passerines typically depart and arrive earlier than females for spring migration to establish breeding territories, and males may also depart slightly earlier than females in autumn [29]. Juvenile males might also be expected to utilise their time differently to females from their very first movements. For example, juvenile males may use their post fledging period prior to autumn migration to explore for future breeding territories [30] thus further highlighting the importance of timing in migration and how it differs for the sexes throughout the annual cycle.

Studies that compare juvenile and adult migrations are rare and biased towards larger species [e.g. 31–33], because tracking migration in small birds can currently only be achieved by using archival light-level or GPS tags, or automated radiotelemetry. These tags must be retrieved the following year which can be challenging, but especially in juveniles where apparent survival is normally low [e.g. 34]. A larger problem of using archival tags arises because they only sample and return data from successful individuals that return to the study site, but where and why unsuccessful birds die is crucial to disentangle the effects of differential survival from learning and experience. Nevertheless, if successful first migrations emerge through differential survival, which could reduce variability in timing, routes, stopovers and non-breeding locations, then we would expect these migration parameters for juveniles on their first migration to be similar to those on their migration following year (i.e. their first migration as an adult), because all surviving juveniles would repeat their first successful migration. This can be investigated by comparing the repeatability (i.e. individual consistency) of migration parameters between juveniles’ first migrations as naive individuals and their subsequent migrations as experienced adults, and between the migrations of adults across two consecutive years. If experience and individual improvements occur as juveniles modify their timing or non-breeding locations for subsequent migrations then we would expect age-related differences between years—i.e. we would expect migration parameters between first migrations of juveniles and their subsequent migration as adults to have lower repeatability than for migrations of adults in two consecutive years because experienced individuals may be less likely to alter their migration parameters.

Here we use light-level geolocator data from archival tags to compare migration timing, deviation of migration from the most direct route, and repeatability of timing and non-breeding locations between juvenile and adult Cyprus wheatears *Oenanthe cypriaca*. We predicted in terms of migratory timing that: juveniles would depart and arrive later than adults for both autumn and spring migration, that males would depart and arrive prior to females in spring, and that duration of migration (the period between migration departure and arrival) would be greater for juveniles. We predicted that migration routes would be less direct for juveniles than adults in autumn, which is typically direct for adult Cyprus wheatears [35]. In terms of repeatability, we predicted that: autumn and spring departure timing, and non-breeding locations would be repeatable for individual adults between two consecutive years, but not for juveniles between their first migration and subsequent adult migration, as juveniles may move at a greater scale post migration as they seek out suitable non-breeding territories, whereas adults migrate directly to familiar areas.

## Methods

### Study site and capture of birds

Cyprus wheatears are small obligate migrant passerines where adults likely migrate non-stop from their breeding range in Cyprus to eastern sub-Saharan Africa, a 2200–3000 km flight (straight-line distance) in ~60 hours [36]. Autumn migration is relatively direct for adults, while spring migration includes an eastern detour that crosses the Red Sea [35].

Light-level geolocator tags were fitted to Cyprus wheatears each year between 2017 and 2019 at the National Forest Park of Troodos, Cyprus (NFP of Troodos; 34°56′11″N, 32°51′48″E). We classed juveniles as birds in their first annual cycle that includes one full round trip before being classed as an adult from their second year onwards. We tagged 241 individual juveniles, 32 of which were retagged a second time (i.e. a year later with a new tag, and classed as an adult for this second migration), and one a third time (i.e. two years later). We tagged 88 individual adults (i.e. birds identified as adults at first capture), 24 of which were retagged a second time, and six a third time. Birds were captured throughout the breeding season from May through to August using mist nests and spring traps in combination with conspecific playback. Each bird was aged and sexed using plumage characteristics [37, 38], and individually identified with a unique combination of four rings, which included three colour-rings and a metal ring provided by BirdLife Cyprus. An additional 141 adults and 166 juveniles were colour-ringed but untagged, for analysis of tag effects on return rates.

We fitted geolocators following methods described in Patchett and Cresswell [35]. We deployed Biotrack ML6740 Mk6 geolocators with 5 mm light stalks fixed at an angle of 45° and used legloop harnesses made from 0.8 mm diameter transparent elastic cord [39]. Twelve of the tags were Biotrack fLight tags with no light stalk and were attached in the same way (note that none of the birds carrying these tags were included in repeatability analyses). The mean combined weight of the harness and Biotrack Mk6 tag was 0.60 ± 0.06 g (mean ± 1 x SD) whilst the mean bird mass was 16.0 ± 1.1 g (mean ± 1 x SD) (range: 3.0–4.7% of the bird’s weight). The mean combined weight of the harness and fLight tags was 0.35 ± 0.02 g (mean ± 1 x SD) whilst the mean bird mass was 16.2 ± 1.2 (mean ± 1 x SD) (range: 2.0–2.4% of the bird’s weight). Harnesses were attached to geolocators prior to fitting to birds and the fitting process took approximately one minute. Adult and juvenile birds appeared to quickly resume normal behaviour on release.

Overall return rates (i.e., birds resighted the following year) of tagged adult birds (46.8%) were not significantly different to untagged adults (52.4%): X^2^ = 0.29, P = 0.59, and return rates of tagged juveniles (23.7%) were not significantly different to untagged juveniles (26.5%): X^2^ = 0.38, P = 0.54. We recaptured 75 of 137 resighted tagged birds, with 68 geolocators providing at least autumn departure data (juvenile male = 19 tags; juvenile female = 15 tags; adult male = 12 tags; adult female = 22 tags). Eleven of these birds were tracked for two consecutive years, with an additional twelfth bird tracked for three years.

### Geolocator data analysis

Raw data were downloaded using BAStrack decompressor software (British Antarctic Survey, Cambridge, UK) and we adjusted for clock drift, assuming that any drift was linear. Further processing and analysis was carried out in R [40]. Twilights were defined using the BAStag R package [41] with a threshold of 2. Outliers that were likely caused by shading were identified using the LoessFilter function in the R package Geolight [42] with K = 5 interquartile ranges. The number of detected outliers varied between 1 and 26 (median = 5) across the 68 tags and were removed from analysis. Summer sun elevation angles (SEAs) were obtained over the breeding period where birds were at a known location (i.e. the study site in Troodos) resulting in SEAs ranging from -5.09 to -2.70 degrees. Coordinates were produced for each twilight using the ‘coord’ function in the Geolight package using the mean summer SEA (-3.97 degrees). Latitude estimates were ignored for two weeks either side of the spring and autumn equinoxes because they are unreliable due to the lack of variation in daylength with latitude over this period. We estimated a single non-breeding location based on the median latitude and longitude coordinates between December to January where previous work has shown Cyprus wheatears to occupy a single non-breeding site [36], except in three cases where tags failed before that period, and in one fLight tag where light data was unusable during this period. Timing of migration was estimated by visual identification of sudden and then sustained changes in plots of sunrise and sunset times. Note that the battery life of the tags varied and hence sample sizes for each migration parameter differ from the total number recovered.

### Statistical analysis

#### Migration timing

We tested for differences in migration timing using linear mixed models (LMMs) in the nlme R package. We included *sex*, *age* (individuals were classed either juvenile or adult) and *year* as fixed effects with *individual* as a random intercept. Tag type and tag batch were assumed to not affect results and were not included analyses. We tested the interaction between *sex* and *age* and found support for its exclusion based on AICc score.

There were two outliers in the autumn migration duration data, and so we ran models with and without these outliers. We found no material difference in results with or without the outliers included and present results with outliers removed and include results with outliers included in the supplementary material.

#### Deviation from the direct migration route

We tested for the degree of deviation from the direct migration route in both autumn and spring by calculating the median longitude during migration (the period between migration departure and arrival) and comparing it to the longitude at the mid-point between the breeding grounds in Troodos and the respective estimated non-breeding location for each bird. If migration routes are direct in autumn (note that great-circle and rhumb line routes are virtually identical in this study system), then we expect the median longitude during migration and the mid-point longitude to be similar. We predicted that adult migration would be more direct than for juveniles in autumn, and hence the difference between the median longitude during migration and the mid-point longitude would be greater for juveniles. We used LMMs to test for differences between adults and juveniles in deviation from the direct route, including *age* and *year* as fixed effects with *individual* as a random intercept. All results report the mean plus or minus one standard error unless stated otherwise. Model fit was assessed by visual inspection of residuals plotted against fitted values and quantile plots.

#### Repeatability

When we tested for repeatability in juveniles, we compared migration parameters from their first year with those from their subsequent year. We tested repeatability of autumn and spring migration departure and arrival timing, and the longitude and latitude of non-breeding locations between years for individuals on their first and second annual migrations (n = 7 and referred to as juveniles), and for individual adults in two consecutive years (n = 6). For non-breeding estimates we calculated the median longitude and latitude during a two-month period (December-January) when adult Cyprus wheatears likely remain at a single location [36]. Note that one tag fitted to a juvenile failed during December and so we excluded this data point when we tested repeatability of non-breeding longitude and latitude. We tested repeatability using the *rpt* function in the rptR package [43]. This uses a linear mixed model framework where the groups compared for repeatability are specified by a random effect (i.e. individual as a random intercept for our study). Confidence intervals were estimated within the *rpt* function by running 999 bootstraps. Significance of repeatability was estimated by a likelihood ratio test within the *rpt* function. We calculated repeatability for females only in the models that tested spring departure and arrival timing because males depart and arrive before females and the sample sizes were n = 1 male for spring departure, and n = 2 for spring arrival. We used the *distHaversine* function in the geosphere package [44] to represent the difference between years in degrees longitude as distance in km for biological context. As with similar studies, our sample size is low for calculating repeatability [e.g. 12, 45, 46] and we approach our results with caution.

High repeatability can result from both large variation among individuals and from high levels of intra-individual consistency, i.e. it shows individual consistency relative to other individuals, thus interpreting repeatability must include consideration of population variability [47]. We described population-level variation in autumn departure timing by calculating the mean, standard deviation and median of the difference between the earliest autumn departure timing from all subsequent autumn departures. We repeated this for autumn arrival and spring departure and arrival, but in spring we calculated the differences from earliest departure or arrival by sex because males depart and arrive before females. We also calculated the range between the minimum and maximum value for each event as a further descriptor of population level variation. Note that we pooled observations from each year of the study when describing population level variation and provide these results as supplementary material.

#### Juvenile movement on the non-breeding ground after migration

If we assume that adults remain stationary at their non-breeding location (or locations) [24, 25, 27, 48], or are just less mobile on the non-breeding grounds than juveniles in their first non-breeding season, then the difference in average non-breeding location estimates between adult and juvenile birds should indicate the scale at which juveniles sample to find their eventual adult non-breeding location(s). If juveniles move more than adults on non-breeding grounds as they sample and explore, then repeatability of non-breeding locations should be higher for adults than for juveniles—with the reasonable assumption that there are no biases in habitat usage between adults and juveniles that could introduce differential geolocator location error between the two groups.

We calculated the difference (distance) in non-breeding locations between years for individuals tracked for two years, and then tested for differences between adults and juveniles in these values using a simple linear model. We assumed that adult Cyprus wheatears remained predominantly in a single territory between December and January as in a previous study on this species [37], and as with similar small migratory species that are territorial and show high site fidelity on non-breeding grounds [24, 25, 27, 48]. We predicted that the difference in distance between non-breeding locations in year 1 and 2 would be greater for juveniles if they disperse to find a suitable non-breeding site after arriving on the non-breeding grounds, whilst adults return directly to a previously occupied non-breeding site.

### Ethics statement

Bird capture and colour-ring marking was carried out with permits and permission through Birdlife Cyprus and the Game and Fauna Service of Cyprus. This research was approved by the School of Biology Ethics Committee, University of St Andrews: SEC17005.

## Results

### Timing

**Fig 1:**
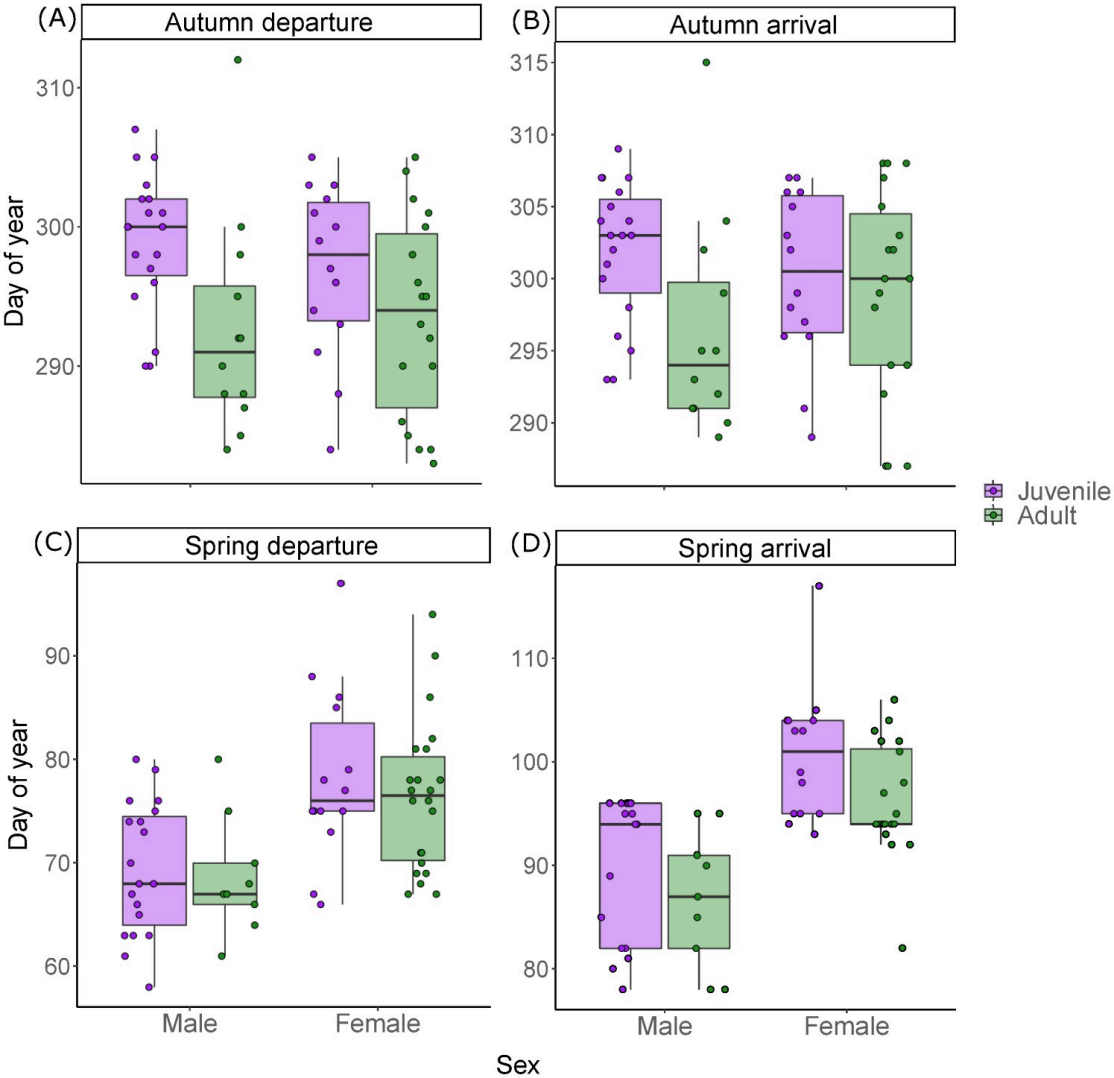
(A-B) Autumn and (C-D) spring migration departure and arrival timing for adult and juvenile Cyprus wheatears. Timing events are estimated from light-level geolocator data. Geolocator tags were deployed in 2017, 2018 and 2019.

Controlling for sex and year, juveniles departed (4.6 ± 1.6 days, t_8_ = 2.82, p = 0.02; R^2^_marginal_ = 0.18, *R*^2^_conditional_ = 0.23) significantly later than adults in autumn (Fig 1), but there was no significant difference in arrival (3.3 ± 1.6 days, t_7_ = 2.0, p = 0.08; *R*^2^_marginal_ = 0.12, *R*^2^_conditional_ = 0.15). There was no significant difference in spring migration departure dates between juveniles and adults (1.4 ± 1.8 days, t_7_ = 0.78, p = 0.46; *R*^2^_marginal_ = 0.26, *R*^2^_conditional_ = 0.64), but juveniles arrived significantly later than adults (4.0 ± 1.7 days, t_6_ = 2.45, p = 0.049; *R*^2^_marginal_ = 0.43, *R*^2^_conditional_ = 0.43) (Fig 1).

Controlling for age and year, there was no significant difference between autumn departure (-0.69 ± 1.7 days, t_50_ = -0.41, p = 0.68) and arrival (0.1 ± 1.6 days, t_51_ = 0.05, p = 0.96) dates of male and female Cyprus wheatears; in spring however, males departed (8.3 ± 2.0 days, t_52_ = 4.2, p < 0.001) and arrived (10.0 ± 1.7 days, t_48_ = 5.82, p < 0.001) significantly earlier than females.

Autumn migration took 3.2 ± 1.6 days, with no significant difference between adults and juveniles, after controlling for sex and year (-0.3 ± 0.4 days, t_7_ = -0.69, p = 0.51; *R*^2^_marginal_ = 0.01, *R*^2^_conditional_ = 0.01), or between males and females, after controlling for age and year (0.16 ± 0.4 days, t_47_ = 0.37, p = 0.71). Spring migration was longer in duration than autumn migration, taking 20.2 ± 5.7 days, again with no significant difference between adults and juveniles (2.6 ± 1.3 days, t_5_ = 1.94, p = 0.11; *R*^2^_marginal_ = 0.04, *R*^2^_conditional_ = 0.04), or between males and females, after controlling for age and year (0.32 ± 1.6 days, t_48_ = 0.19, p = 0.84).

### Deviation from the direct migration route

**Fig 2:**
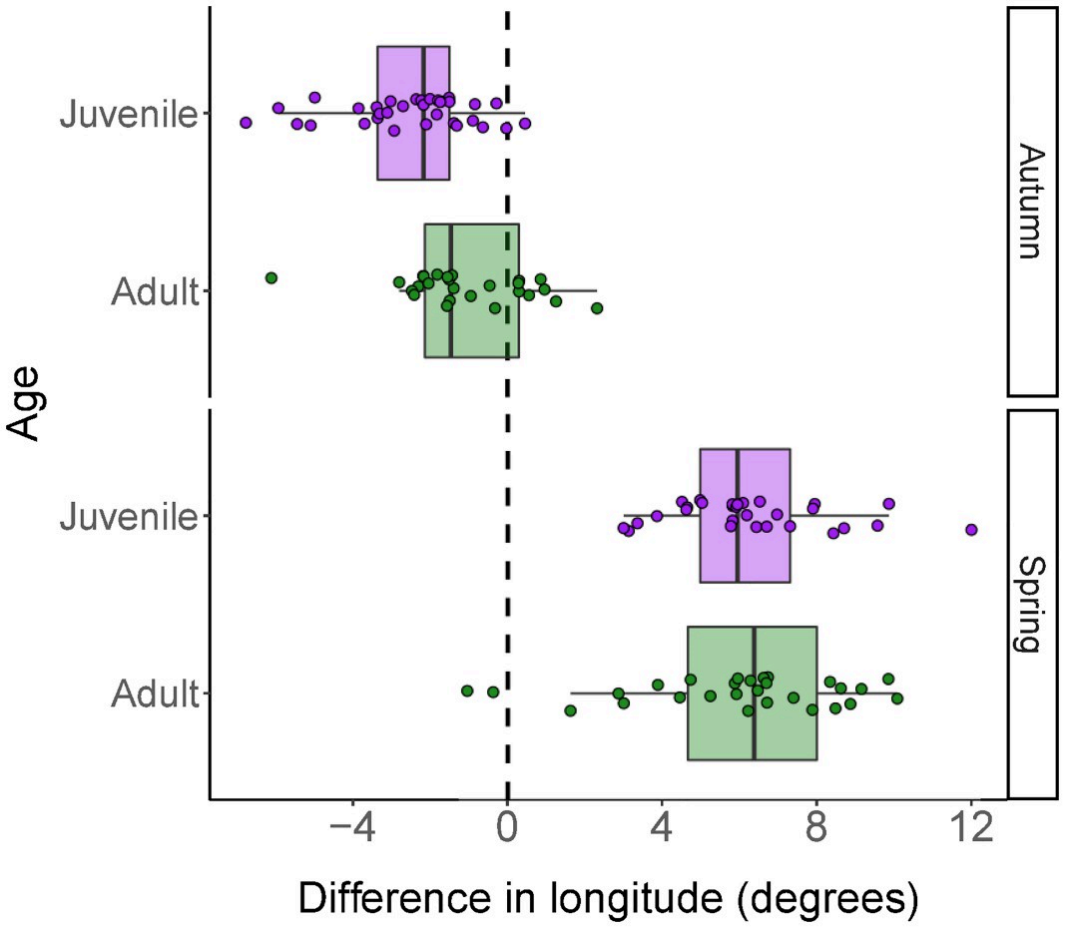
Juvenile Cyprus wheatear migrations are less direct than adults, but only in autumn. Differences in longitude (degrees) between the median longitude during migration and the longitude at the mid-point between the breeding grounds in Troodos, Cyprus, and non-breeding locations for autumn and spring migrations. Positive values reflect deviations to the east. Data were estimated from light-level geolocation data. Geolocator tags were deployed in 2017, 2018 and 2019.

**Fig 3:**
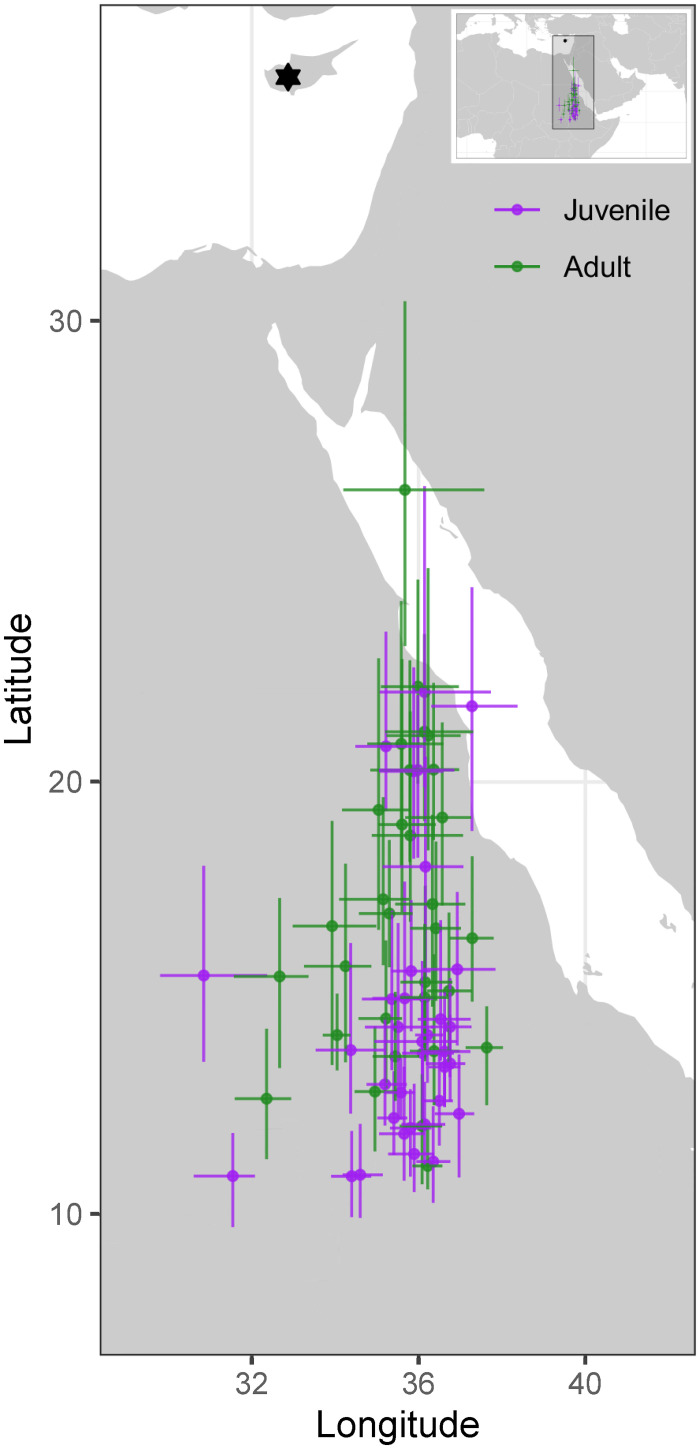
Non-breeding locations of adult and juvenile Cyprus wheatear. Locations are median location during December-January. Error bars show inter-quartile range. The black star shows location of the breeding ground field site in Troodos, Cyprus.

In autumn, the median longitude of juveniles during migration was significantly further away (1.42 ± 0.46 degrees longitude, t_6_ = 3.10, p = 0.02; *R*^2^_marginal_ = 0.16, *R*^2^_conditional_ = 0.26; Fig 2) from the midpoint longitude between breeding ground and non-breeding locations (Fig 3) than that of adults (257 ± 29 km, n = 33 for juveniles; and 161 ± 24 km, n = 25 for adults). However, there was no difference between juveniles and adults in spring (-0.54 ± 0.64 degrees, t_5_ = -0.84, p = 0.44; *R*^2^_marginal_ = 0.10, *R*^2^_conditional_ = 0.10) (638 ± 39 km, n = 29 for juveniles and 607 ± 49 km, n = 28 for adults) (Fig 2), with both following a previously described detoured route east of the autumn route [35].

### Repeatability

**Fig 4:**
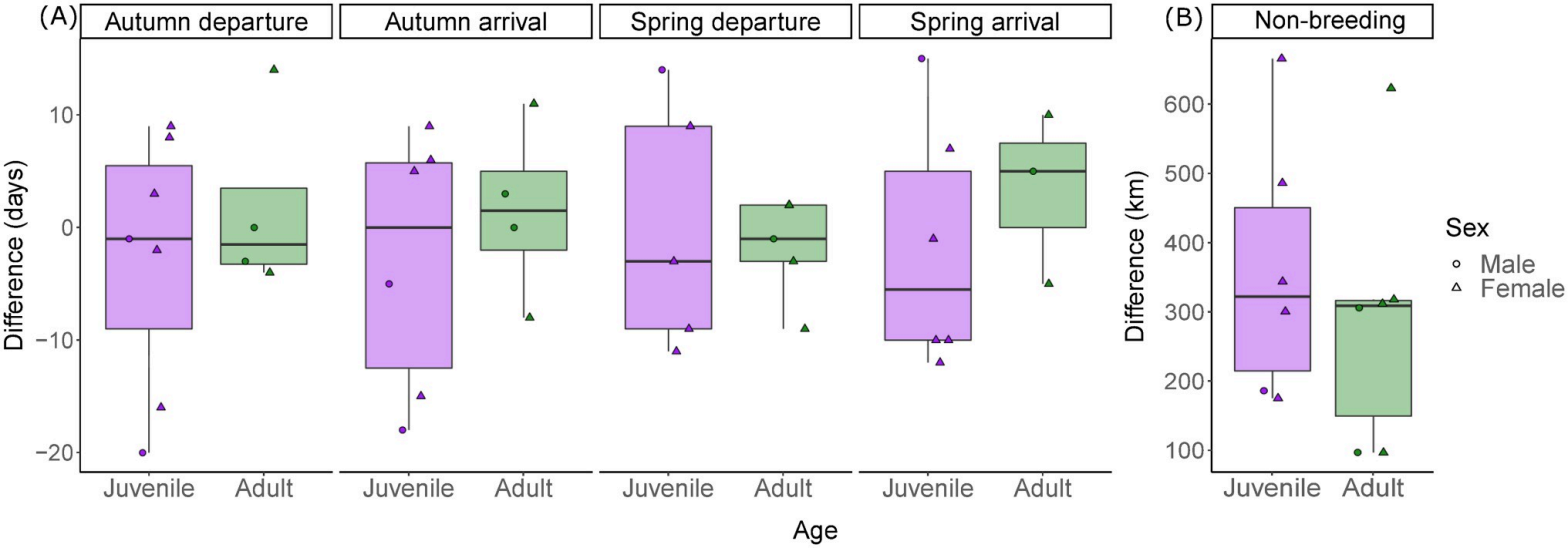
Intra-individual variation in (a) migration timing and (b) distance between non-breeding location for adult and juvenile Cyprus wheatears. Differences are between two consecutive years from light-level geolocation data. Timing differences (a) show the direction of the difference, i.e. whether the difference is earlier or later between year one and two.

Spring departure timing had high and significant repeatability for adults (n = 4 individuals, n = 2 years, r = 0.83, p = 0.028) but not juveniles (n = 5 individuals, n = 2 years, r = 0.35, p = 0.50) (Table 1). Autumn arrival and departure, and spring arrival were neither repeatable for adults nor juveniles (see Table 1). Depending on the individual, migration timings were either earlier or later in the second year of tracking for both juveniles and adults (Fig 4A). See S1 Table in S1 File for description of population variation in migration parameters.

**Table 1:** Repeatability (r) of migration timing and non-breeding longitude and latitude for juvenile and adult Cyprus wheatears. Sample size is the number of individuals and each individual was measured in two consecutive years. The repeatability score r ranges from 0 to 1 with a score of 1 indicating high similarity between years. The p-value tests if r is significantly different from zero. Italicized numbers are significant.

Migration event	Age	Repeatability				
		n	r	SE	CI	p
Autumn Departure	Adult	4	0.348	0.311	0–0.911	0.299
Autumn Departure	Juvenile	7	0	0.207	0–0.682	0.5
Autumn Arrival	Adult	4	0.223	0.301	0–0.889	0.428
Autumn Arrival	Juvenile	6	0	0.227	0–0.727	0.999
Spring Departure	Adult	4	*0*.*827*	*0*.*254*	*0–0*.*980*	*0*.*028*
Spring Departure	Juvenile	4	0.387	0.317	0–0.897	0.251
Spring Arrival	Adult	2				
Spring Arrival	Juvenile	5	0	0.226	0–0.746	0.999
Non-breeding longitude	Adult	6	*0*.*706*	*0*.*257*	*0–0*.*938*	*0*.*033*
Non-breeding longitude	Juvenile	6	0	0.215	0–0.688	0.5
Non-breeding latitude	Adult	6	*0*.*663*	*0*.*267*	*0–0*.*927*	*0*.*048*
Non-breeding latitude	Juvenile	6	0.593	0.279	0–0.908	0.08

Non-breeding longitudes and latitudes had high and significant repeatability between years for adults (longitude: mean difference 172 ± 86 km, n = 6 individuals, n = 2 years, r = 0.71, p = 0.033; latitude: mean difference 146 ± 73 km, n = 6 individuals, n = 2 years, r = 0.66, p = 0.048) but not for juveniles between their first and second year (longitude: mean difference 261 ± 99 km, n = 6 individuals, n = 2 years, r = 0, p = 0.5; latitude: mean difference 221 ± 84 km, n = 6 individuals, n = 2 years, r = 0.59, p = 0.08) (Table 1; Fig 4B). The distance between non-breeding locations in year 1 and year 2 was not significantly different between adults and juveniles (-67 ± 110 km, t_(10)_ = -0.61, p = 0.55; Fig 4B).

## Discussion

We found that juveniles departed later than adults in autumn, but departed for spring migration at a similar time as adults. They arrived on the breeding grounds after adults, yet there was no difference in migration duration between adults and juveniles. Juveniles deviated from the direct route more than adults in autumn, but there was no difference between adults and juveniles in spring with both groups following a detoured route [35]. Together these findings suggest that age-related changes in migration occur within individuals during the first annual cycle. In longer-lived species, the development and improvement of migratory behaviour occurs gradually over several years [e.g. 31], but in shorter-lived species such as the Cyprus wheatear, we suggest that process may be more rapid. We note that Cyprus wheatear migration is relatively short compared to other obligate migratory passerines, so age-related differences in migration are likely to be stronger in species with multiple stopovers and longer migrations. We also suggest that flexibility in migration timing of adults and juveniles may allow for some adjustment to advancing spring phenology. But we note our methods do not consider the role of differential survival because we only sample successful birds.

### Timing

First migrations of juvenile Cyprus wheatears differed in timing compared to experienced birds. Juveniles departed for autumn migration later than adults, but the overall duration of this first migratory movement was similar to adults. This contrasts with previous work based on ringing recoveries that found migration speed to be greater in adults [49, 50]. The similarity in duration perhaps reflects that the main migratory movement in autumn for Cyprus wheatears occurs in a single flight [36] and is aided by supportive winds [35]. In comparison to other species, this migration is uncomplicated and direct, with no evidence of stopovers of more than a few hours [36], reducing the possibilities for differences in stop-over duration [21, 51] or number of stops [18] between juveniles and adults to accumulate. For example, no age-related differences were found in autumn migration of Eleonora’s falcon *Falco eleonorae* until after they had crossed the Sahara, where juveniles then migrated less directly and had longer stopovers than adults [21]. It is possible, however, that barrier crossings result in lower survival probabilities for juveniles than adults [52] so that our observed crossing durations appear similar between experienced and first-time migrants only because we have data from the survivors (and see Blackburn et al. [53] for similar observations of Sahara crossings by adult and juvenile whinchats *Saxicola rubetra* in spring). We note that even though autumn routes were more direct for adults than juveniles, there was no difference in duration as might be expected. We suggest that the additional migration distance is relatively small and is likely to be within the precision that geolocators allow for timing differences to be detected, i.e. positions can be estimated only every 12 hours and the potential additional flight time for juveniles on their less direct route is likely to be less than this.

The autumn migration departure timing difference between juvenile and adult Cyprus wheatears is common among long-distance migrant passerines [54]. But even though juvenile Cyprus wheatears departed after adults on average, the distributions of departure times overlap considerably between the sex and age classes so that many juveniles and adults depart at similar times. Thus, we cannot exclude that some juveniles may use information gained by observing conspecifics to inform them when to depart for their first migratory flight. But because the autumn migration routes were less direct in juveniles than in adults, once they depart for their first migration, they presumably have fewer opportunities to acquire information from experienced individuals, so that social information is incidental rather than a crucial part of their first migration.

We observed protandrous migration in spring which is typical for many birds [29]. The departure dates for spring migration were similar between adult and juveniles for both males and females, but juveniles on average arrived at their breeding grounds later. This timing difference was only weakly reflected in the duration of the period between departure and arrival, where, although greater for juveniles, it was not significantly different (p = 0.1, n = 29 juveniles and n = 29 adults). That juveniles began their main spring migratory movement at a similar time to adults but arrived later may be due to spring migration being longer and less direct compared to their autumn migration [35]. This longer spring migration is likely to have included at least one stopover, which may have increased timing differences between juveniles and adults [21], or juveniles may have stopped more frequently than adults [18]. We cannot determine definitively if and where stopovers occurred though, because spring migration included the spring equinox and so latitude estimates are unreliable [55]. This also suggests that age-related differences in migration parameters are likely to be stronger in species that have longer migrations with more stopovers. We were also unable to determine differences in pre-migratory fuelling which may have differed in its timing between adults and juveniles [20].

We found evidence for spring departure timings being repeatable for adults, but we caution on the uncertainty in all repeatability results due to small sample sizes. Population variability in spring departure spanned a 27-day period for adults and 31 days for juveniles (see S1 Table in S1 File) and adults tracked over two years departed on average ~3 days apart between years, resulting in spring migration timing for adults being more repeatable than at other stages in the migratory schedule and compared to juveniles in spring (~9 days between years). High repeatability of spring migration departure timing has been observed in other passerines [46, 56, but see 45] and suggests that it is under stronger endogenous control than in autumn. Spring departure for juveniles was earlier or later in the second year depending on the individual, rather than being directional (Fig 4A). This difference may be a result of juveniles improving their departure timing through experience [57], or juveniles may respond less optimally to cues such as photoperiod than they do as adults [18]. That juveniles adjusted their migration timing between years indicates a degree of flexibility that may confer some capacity to adapt to climate-change-driven shifts in spring phenology [11, 57]. Similarly, some juveniles modified their second autumn migration to be earlier, whilst others migrated later, suggesting flexibility that may allow them to wait for suitable departure conditions. But we again note caution in our repeatability results due to the low sample size.

### Route directness

All birds followed an eastern detour for their spring migration that is likely to provide more wind assistance than if they returned directly following the autumn route [35]. Juveniles migrating in spring are still naive to this detour yet first-time migrants that successfully returned to the breeding grounds still took this route. How juveniles decided to follow this detour is unknown. Our data do not exclude the role of social information influencing departure and route decisions of juveniles, indeed distributions of departure times were similar in spring between juveniles and adults, and all else being equal we expect that juveniles should use information and cues from experienced birds to reduce uncertainty [58]. However, the mechanism and practical efficiency of this has not yet been clearly established in any migrant passerine species. Route choice is likely to have an endogenous component [59] and we cannot exclude that some individuals may have taken alternative routes that were unsuccessful so that the detour emerges through differential survival. These ideas are not mutually exclusive, and it is possible that these processes operate together to shape the seasonally different migration routes of Cyprus wheatears.

### Non-breeding locations

Studies that attempt to identify the scale of dispersal on the non-breeding grounds are limited [e.g. 28], yet understanding dispersal and the scale that it occurs over is an important part of identifying how resilient populations might be to habitat change. This is especially true in migratory species because juvenile dispersal likely occurs on both the breeding and non-breeding grounds, and both locations may be subject to habitat change. Juveniles on their first migration are moving to an unknown non-breeding location, whereas adults are likely to be returning to previously used non-breeding sites [24, 25, 27]. This might explain why adult Cyprus wheatear migrations were more direct than juveniles’ [e.g. 21, but see 19] because adults may be more likely to adjust their course toward their known destination [23, but see 59]. After arriving at the non-breeding grounds, juveniles may then disperse locally to find a suitable territory that they then return to directly the following year [60], and that we found high repeatability of non-breeding longitudes and latitudes for adults but not for juveniles supports this idea.

Assuming that adults remain in a single non-breeding location during December to January, and that the difference between years in adult non-breeding location represents geolocator error (or error plus adult movement), then the difference between non-breeding locations for adults in consecutive years and for juveniles in their first and second year suggests an increase in movement for juveniles of 67 ± 110 km compared to adults, reflecting the approximate scale of their post-migration dispersal. We note that this difference was not significant: these results can only be confirmed with accurate tracking where locations can be resolved at the kilometre scale, nevertheless, the results show that if any adjustment in location during a juvenile’s first year occurs, it is likely only over this relatively small scale.

### Prospectus

The development and improvement of migration with age is likely to be influenced by changes in experience that we have shown here, but also selection against unsuccessful migrants (i.e. differential survival) [31]. Our study is biased toward successful migrants. The routes, timings and non-breeding areas used by unsuccessful small long-distance migrants remain unknown, and this information is important in fully understanding non-breeding dispersal, the evolution of migration routes, and population dynamics. It is possible that some unsuccessful juveniles were displaced as is common in other species during autumn migration [2], so that they end up in unsuitable non-breeding grounds that reduce probability of survival. This stochasticity in resultant non-breeding locations of first-time migrants has been argued to be adaptive where the quality of non-breeding habitat is unpredictable over inter-generational timescales [8, 61]. Knowing the fate of unsuccessful migrants remains the key next step in understanding processes in the ontogeny of migration and how adaptable migrants are to change [4].

To understand the relative effects of differential survival and experience on migration we need to track individuals with high resolution satellite data transmitters, rather than archival tags. This will allow us to identify when and where during the annual cycle mortality occurs [4]. It will also allow fine-scale examination and comparison of movement patterns on the non-breeding ground between first time and experienced birds, and this can only be achieved by tracking juveniles from the breeding ground to encompass the complete non-breeding period. Nevertheless, our results show that differences between juvenile and adult autumn migration departure timing and route are not present in spring migration, demonstrating that age-related changes in migratory behaviour begin to occur within the first annual cycle.

## Supporting information

S1 File (PDF)
