# Supplementary material for: Age-related changes in migratory behaviour within the first annual cycle of a passerine bird
Source: PLoS One. 2022 Oct 19;17(10):e0273686. doi: 10.1371/journal.pone.0273686 (PMC9581414; doi:10.1371/journal.pone.0273686)
Supplement: S1 File — (PDF) [file pone.0273686.s001.pdf]

Supplementary Material

Age-related changes in migratory behaviour within the first annual cycle of a passerine bird

Robert Patchett[1], Alexander N. G. Kirschel[2], Joanna Robins King[1], Patrick Styles[1] and Will Cresswell[1]

[1]Centre for Biological Diversity, University of St Andrews, St Andrews, Fife KY16 9TH, UK.

[2]Department of Biological Sciences, University of Cyprus, PO Box 20537, Nicosia 1678, Cyprus.

Supplemental Results

For two adult females in 2018, the period between departure and arrival for autumn migration was 12 and 15 days, respectively. Autumn migration took 3.5 ± 2.5 days (mean ± SD), with no significant difference between adults and juveniles after controlling for sex and year (-0.9 ± 0.6 days, $t_7 = -1.48$, $p = 0.18$), or between males and females after controlling for age and year (-0.4 ± 0.6 days, $t_{49} = -0.58$, $p = 0.56$).

Table S1. Population variation of migration timing and non-breeding longitudes and latitudes for adult and juvenile Cyprus wheatears.

			Population variability		
Migration event	Age	n	Mean ± SD	Median	Range
			Days		
Autumn Departure	Adult	30	10.1 ± 7.4	9	29
Autumn Departure	Juvenile	33	14.1 ± 5.5	16	23
Autumn Arrival	Adult	30	10.9 ± 7.4	11.5	28
Autumn Arrival	Juvenile	33	12.2 ± 5.3	14	20
Spring Departure	Adult	31	8.9 ± 6.9	9	27
Spring Departure	Juvenile	33	11.8 ± 7.2	10	31
Spring Arrival	Adult	29	12.6 ± 6.3	12	24
Spring Arrival	Juvenile	30	9.8 ± 7.0	10.5	24
			Degrees longitude		
Non-breeding longitude	Adult	34	4.8 ± 1.3	5.1	6.4
Non-breeding longitude	Juvenile	31	3.2 ± 1.7	3.5	5.1
Non-breeding latitude	Adult	34	3.7 ± 3.2	2.9	11.1
Non-breeding latitude	Juvenile	31	5.8 ± 3.4	5.5	15.3
